# Neurophysiological correlates of clinical improvement after greater occipital nerve (GON) block in chronic migraine: relevance for chronic migraine pathophysiology

**DOI:** 10.1186/s10194-018-0901-z

**Published:** 2018-08-20

**Authors:** Alessandro Viganò, Maria Claudia Torrieri, Massimiliano Toscano, Francesca Puledda, Barbara Petolicchio, Tullia Sasso D’Elia, Angela Verzina, Sonia Ruggiero, Marta Altieri, Edoardo Vicenzini, Jean Schoenen, Vittorio Di Piero

**Affiliations:** 1 Headache Centre & Neurocritical Care Unit. Department of Human Neurosciences, Sapienza – University of Rome, Viale dell’Università 30, 00185 Rome, Italy; 2 Molecular and Cellular Networks Lab. Department of Anatomy, Histology, Forensic medicine and Orthopaedics, Sapienza – University of Rome, Rome, Italy; 3 Rita Levi Montalcini Department of Neuroscience, Città della Salute e della Scienza, Turin, Italy; 4 Department of Neurology, Fatebenefratelli Hospital, Rome, Italy; 5 Headache Group, Department of Basic and Clinical Neuroscience, King’s College London, and NIHR-Wellcome Trust King’s Clinical Research Facility, Wellcome Foundation Building, King’s College Hospital, London, SE5 9PJ UK; 6 Department of Neurology, University of Perugia, Perugia, Italy; 7 Headache Research Unit. Department of Neurology, University of Liège, Citadelle Hospital, Liège, Belgium; 8 University Consortium for Adaptive Disorders and Head pain – UCADH, Pavia, Italy

**Keywords:** Habituation, Serotonin, Chronic migraine, Predictors, Plasticity

## Abstract

**Background:**

Therapeutic management of Chronic Migraine (CM), often associated with Medication Overuse Headache (MOH), is chiefly empirical, as no biomarker predicting or correlating with clinical efficacy is available to address therapeutic choices. The present study searched for neurophysiological correlates of Greater Occipital Nerve Block (GON-B) effects in CM.

**Methods:**

We recruited 17 CM women, of whom 12 with MOH, and 19 healthy volunteers (HV). Patients had no preventive treatment since at least 3 months. After a 30-day baseline, they received a bilateral betamethasone-lidocaine GON-B of which the therapeutic effect was assessed 1 month later. Habituation of visual evoked potentials (VEP) and intensity dependence of auditory evoked potentials (IDAP) were recorded before and 1 week after the GON-B.

**Results:**

At baseline, CM patients had a VEP habituation not different from HV, but a steeper IDAP value than HV (*p* = 0.01), suggestive of a lower serotonergic tone. GON-B significantly reduced the number of total headache days per month (− 34.9%; *p* = 0.003). Eight out 17CM patients reversed to episodic migraine and medication overuse resolved in 11 out of 12 patients. One week after the GON-B VEP habituation became lacking respect to baseline (*p* = 0.01) and to that of HV (*p* = 0.02) like in episodic migraine, while the IDAP slope significantly flattened (*p* < 0.0001). GON-B-induced reduction in headache days positively correlated with IDAP slope decrease (rho = 0.51, *p* = 0.03).

**Conclusions:**

GON-B may be effective in the treatment of CM, with or without MOH. The pre-treatment IDAP increase is compatible with a weak central serotonergic tone, which is strengthened after GON-B, suggesting that serotonergic mechanisms may play a role in CM and its reversion to episodic migraine. Since the degree of post-treatment IDAP decrease is correlated with clinical improvement, IDAP might be potentially useful as an early predictor of GON-B efficacy.

## Background

CM is a complication of episodic migraine affecting 2% of the general population [1] and most commonly encountered in tertiary headache clinics [2]. It is frequently associated with Medication Overuse Headache (MOH), a secondary headache, which could be interpreted as a comorbidity as well as a marker of poor control of the migraine itself [3, 4]. In the migraine pathology spectrum, chronic migraine (CM) represents the most severe form and its disabling effects are not only related to the physical health of patients but also reverberate on social and economic aspects. The available pharmacological treatments of CM are quite limited. Only a few drug treatments like topiramate [5] and onabotulinumtoxin A [6, 7], have an efficacy proven by randomized controlled trials. Most drugs commonly used in the treatment of CM have been investigated only by small open studies (for a review, see [8]). Management of CM patients is further complicated by poor therapeutic adherence due to the insufficient efficacy of drug therapies and their side effects [9].

Since no predictors of treatment response are available to date, preventive medications are selected by trial and error, taking into account the patients’ clinical profile, drug side effects and the patients’ comorbidities more so than by their efficacy differences. Each drug is commonly recommended for 4–6 months before assessing (in)efficacy and CM patients try on average 4 therapeutic lines with severe patient discomfort and frustration of the doctor. Half of patients interrupt treatment due to lack of efficacy or side effects and only about 9% achieve clinical improvement [10].

Greater occipital nerve blocks with a local anesthetic alone [11, 12] or with a steroid-local anaesthetic combination [13, 14] were found useful for CM with varying effect sizes and durations, but their mechanism of action remains elusive.

Repetition of attacks and failure of preventive therapies produce a longer exposure to headaches that might promote central sensitization and maintain migraine chronicity and possibly refractoriness [8, 15].

Understanding neurophysiological mechanisms of chronification might allow identifying biomarkers able to predict transition and remission from chronic to episodic migraine (EM).

Possible candidates could be markers of brain excitability and responsivity, such as habituation and amplitude-stimulus function of cortical evoked responses (measured by IDAP) [16, 17].

Detailed description of the lack of habituation in migraineurs has been widely reported elsewhere [17, 18]. In EM between attacks, during repeated and unmodified sensory stimulation, cortical evoked responses are characterised by a reduced initial amplitude (lower preactivation level) and a progressive amplitude increase (i.e. hyperresponsivity) instead of a physiological habituation, regardless of the sensory modality, visual, auditory, somatosensory, laser, including cognitive potentials like contigent negative variation or P3 [19]. All evoked potentials, besides laser evoked potentials, normalize during migraine attacks [20]. Interestingly, in CM the electrophysiological pattern is similar to that of ictal EM recordings, suggesting genuine hyperexcitability and supporting the idea that CM is a sort of “never-ending attack” [21, 22]. MOH patients have an intermediate pattern between CM and EM with an increased initial response but a lack of habituation, a pattern that is found in the pre-ictal EM phase [23].

When CM patients improve and revert to EM, they acquire again the interictal electrophysiological profile consisting of a low initial response and a deficient habituation [24].

Intensity Dependence of Auditory evoked Potentials (IDAP) is chiefly modulated by activity in central brainstem-cortical serotonergic projections [25]. In interictal EM IDAP is increased reflecting low serotonergic tone, and correlates with deficient habituation [26]. So far, no IDAP data are available in CM. It is thus not known if IDAP is correlated or not with habituation as in EM nor if it changes when patients revert from CM to EM.

In this study, we aimed to evaluate changes in IDAP before and 1 week after a greater occipital nerve block (GON-B) with a steroid-local anaesthetic combination, as well as clinical changes 4 weeks after the block in CM/MOH patients without preventive therapy. Besides confirming a possible therapeutic effect, our aim was to search for a correlation between such an effect and baseline IDAP or its GON-B induced early change in order to identify a biomarker able to predict the treatment effect.

## Methods

### Subjects

We consecutively recruited 17 female Chronic Migraine (CM) patients with or without Medication Overuse Headache (MOH) (ICHD-III 1.3 and 8.2) in the Headache Clinic of the University Hospital of Rome, Policlinico Umberto I, according to ICHD-III criteria. We included only > 18 years old patients, who had: i) a diagnosis of Chronic Migraine (ICHD-III 1.3); ii) migraine without aura (ICHD-III 1.1) at origin; iii) no spontaneous improvement of migraine burden in the previous 6 months. Twelve of them also qualified for a diagnosis of MOH (ICHD-III 8.2).

Exclusion criteria were other neurological and psychiatric conditions (e.g. epilepsy, cerebrovascular diseases, etc.), a Beck Depression Inventory higher than 11 points, or medical contraindications to receive GON infiltration (e.g. allergies, infections, open skull defect, anticoagulant use), other chronic painful conditions. Prophylactic migraine treatment and anti-depressants were not allowed for at least three months before trial inclusion, nor were acute migraine drugs during minimum 12 h before the neurophysiological recordings.

Healthy volunteers (HV, *n* = 19) of comparable age and gender distribution, without personal or family history of migraine, neurological or psychiatric disease, use of medications acting on brain excitability or serotonin were recruited for comparative electrophysiological recordings.

The local Ethics Committee approved the study and all patients gave their written informed consent to take part in the experiment. The study was conducted in accordance with the Helsinki Declaration.

### Intensity dependence of auditory evoked potentials (IDAP)

Auditory Evoked Potentials (AEPs) were recorded as recommended [27] and implemented elsewhere [28, 29]. AEPs were evoked at four different intensities (60, 70, 80 and 90 dB) in a pseudo-randomized order. For each intensity level, 90 trials were collected with a sampling frequency of 4000 Hz and sweep duration of 400 ms (50 ms before and 350 ms after the auditory stimulus). Traces were filtered offline with a 1-20 Hz bandpass filter. We identified N1 components (the maximal negative deflections between 60 and 150 ms post-stimulus) and P2 (the positive deflections between 120 and 200 ms), and measured N1P2 peak-to-peak amplitudes at each stimulus intensity. IDAP value was calculated as the linear amplitude/stimulus intensity function (ASF) slope (IDAP slope) for block averages (μV/ 10 dB).

### Pattern reversal-visual evoked potentials (PR-VEPs).

PR-VEPs were performed as recommended [27], similarly to the protocol used in our previous study [30].

During uninterrupted stimulation, 250 cortical responses were recorded by a BrainVision preamplifier at a sample a rate of 4000 Hz, and divided in epochs of 300 ms after the stimulus. We identify N1 as the first negative wave occurring around 75 ms from the stimulus, P1 as a positive wave occurring after N1 at around 100 ms, N2 as a negative wave around 145 ms. Cerebral responses were divided in 5 consecutive block and the habituation was calculated as the slope interpolating the average of amplitudes in each block (habituation slope). Negative values reflect habituation (i.e. a decrement of responses over time) whereas positive values indicate lack of habituation (i.e. an increase of responses).

### Anaesthetic block of GON (GON-B)

Bilateral GON blocks were performed with a 21-gauge needle, injecting suboccipitally midway between inion and mastoid, a 3 ml solution of betamethasone sodium phosphate 4 mg (2 ml) and xylocaine 2% (1 ml).

### Study design

**Fig. 1:**
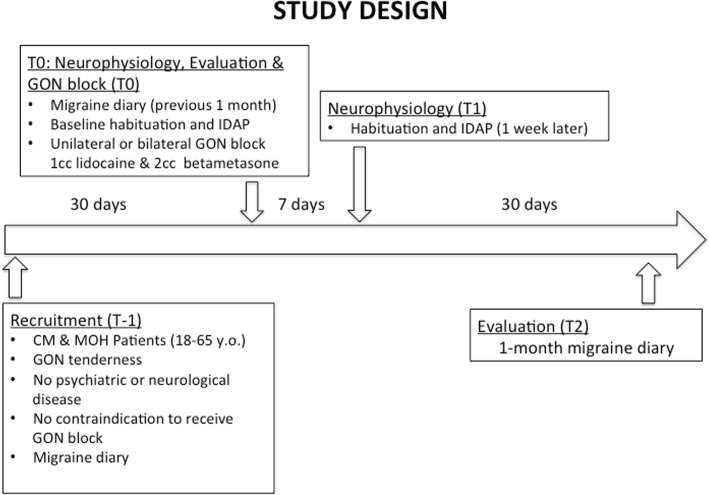
Study design. Patients were recruited at T0. After 1 month of clinical evaluation, we performed neurophysiological recordings (habituation and IDAP) prior to GON anaesthetic block. Early neurophysiological changes were collected after a week and clinical revaluation was planned at 30 days

The study design is shown in Fig. 1. During the 1st visit (T-1), CM patients were enrolled and asked to fill in a 30-day headache diary (pre GON-B), recording number and duration of attacks, as well as headache intensity of and associated symptoms using a 3-point scale from 1 (mild symptoms) to 3 (severe symptoms). Patients also recorded the number of acute medication intake.

At the 2nd visit (T0), clinical data were collected from the diary and PR-VEPs habituation and IDAP were measured. Thereafter, patients received a bilateral GON-B. Patients with acute medication overuse were not detoxified but advised to reduce their consumption of acute drugs.

PR-VEPs and IDAP were repeated after one week ±1 day (T1), and possible adverse effects of GON-B were assessed.

In women, all neurophysiological recordings were performed between day 2–10 of the luteal phase of the menstrual cycle.

Patients were clinically re-evaluated 1 month after GON-B (T2) and the post-treatment diary data were compared to the pre-treatment data.

Blinded investigators analysed the clinical and electrophysiological data (MT) and performed the statistics (AVi).

### Outcome measures and statistics

We tested normal distribution of variables with Shapiro-Wilk’s test. The primary clinical outcome measure was the reduction in number of total headache days (between the pre GON-B and post GON-B month). We also calculated the recovery rate from overuse of acute medication. We used Wilcoxon’s rank test or Fisher’s 2 × 2 contingency tables for clinical variables (non-normally distributed). Neurophysiological variables had a normal distribution, and then baseline values and the changes induced by GON-B of neurophysiological tests were compared between groups with repeated measured ANOVA and Fisher’s LSD test for post-hoc comparison. Since one objective of the study was to identify mechanisms related to migraine chronification and its reversal, CM patients who reversed to EM were labelled as “responders” for further sub-analyses.

We also used repeated-measures ANOVA, with Fisher’s LSD test for post-hoc comparison, to compare temporal changes between pre GON-B and post GON-B in Responders (R) and Non-Responders (NR).

The correlation analysis between neurophysiological and clinical data was performed with Spearman’s rho. Statistical analyses will be performed with STATISTICA 7 (StatSoft, Tulsa, Ok). Group values were represented by means ± standard deviation. Significance level was set at *p* < 0.05 after multiple comparison correction.

## Results and discussion

### Clinical effects of GON-B in chronic migraine

In twelve months, we recruited 20 women with CM who met the study selection criteria. Three patients dropped out since they didn’t report headache diary at T2 visit. Twenty female healthy volunteers (HV) were recruited as controls, one of them dropped out as she missed a scheduled recording. Baseline characteristics of both groups are shown in Table 1. Twelve of 17 patients had also MOH, of whom seven patients overused analgesics, four triptans, one a combination of caffeine, indometacine, prochlorperazine.

**Table 1:** Baseline characteristics of CM patients

Parameters	Patients	Healthy volunteers	*p*-value
Age	32.9 ± 14.5	31.45 ± 13.94	*p* = 0.63
Gender	17 F	19 F	–
N° of headache days per month	24.88 ± 7.22	–	–
Medication Overuse Headache	12 (70%)	–	–
Type of medication overused		–	–
	Triptans: 4 (33%)		
	NSAIDs: 7 (58%)		
	Combination: 1 (8%)		
N° of acute medication per month	16.18 ± 12.31	–	–
Pain intensity (1–3 scale)	1point 0 pts		
	2 points 3 pts	–	–
	3 points 15 pts		
Habituation N1P1 at T0	0.23 ± 0.68	0.26 ± 0.63	*p* = 0.53
Habituation P1N2 at baseline	0.20 ± 0.55	0.08 ± 0.53	*p* = 0.54
IDAP at baseline	0.76 ± 0.95	0.03 ± 0.76	*p* = 0.01*

The star (*) indicates the p level after post-hoc comparison, when appropriate. Otherwise, p level indicated refers to the one obtained by the repeated measures model

**Fig. 2:**
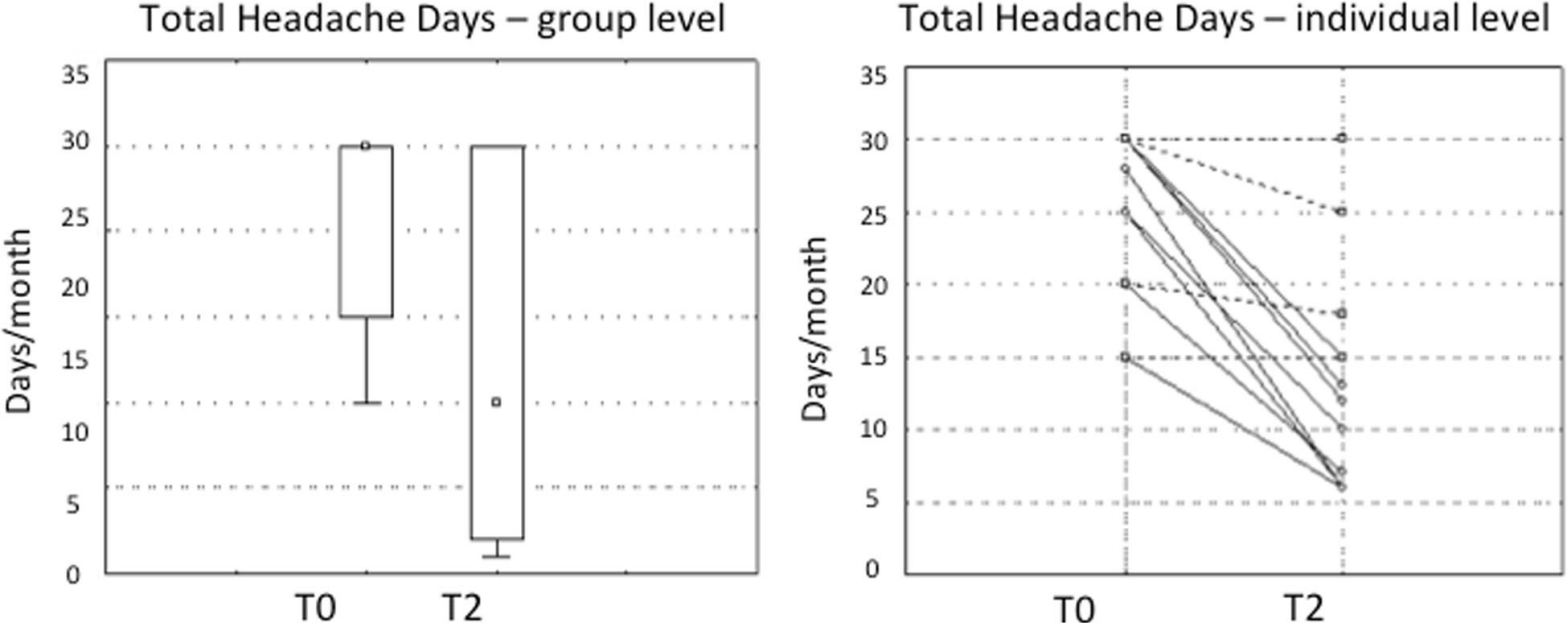
Clinical effect at group and individual level. GON block was effective in reduce the average clincial burden of chronic migraine by 35%. More over we observed that after GON-B the majority of patients who responded reversed to EM. Only one case had a 15 days reduction but passed from 30 days to 15 days of headache remaining within the boundary of chronic migraine. Most of patients, who didn’t respond, had no benefit (five of them are 30 days to 30 days and then superimposed in the graph). Two Responders passed from 15 days to 6 days and are superimposed in the graph. Responders are in dotted, No-responders in continuous line

In the group level analysis, the mean number of headache days decreased after the GON procedure from 24.88 ± 7.22 to 16.59 ± 10.25 (− 34.9%, *p* = 0.003) (see Fig. 2). Eight out 17 CM patients reversed to episodic migraine.

The GON-B had a significant effect also in MOH patients. MOH resolved in 11 out of the 12 MOH patients. One patient, who didn’t respond to GON-B, developed MOH in the month following the procedure. The clinical benefit of GON-B on stopping the medication overuse was significant (Fisher’s 2 × 2 test, *p* = 0.001).

### Neurophysiological changes at baseline and after GON-B between CM patients and HV

We tested baseline differences between HV and CM patients and changes induced by GON-B within each group by using repeated measure ANOVA model, separately for each neurophysiological variable. For N1P1 habituation, the model reported no significant difference in the model neither at baseline between groups nor in each group (HV vs. CM patients) after GON-B (repeated measure ANOVA F(1,34) = 0.40, *p* = 0.53). By contrast, for P1N2 we found a significant result in the model (repeated measure ANOVA F(1,34) = 4.37, *p* = 0.04). After post-hoc comparison, in fact, CM patients didn’t differ from HV for P1N2 at baseline (p = 0.53), but they had a significant impairment of habituation after GON-B (0.20 ± 0.54 vs. 0.55 ± 0.72, *p* = 0.01) so that a T1 the value of habituation was significant lower in CM than in HV (CM: 0.55 ± 0.72 vs. HV: 0.08 ± 0.53, *p* = 0.03, *p* = 0.02).

As well, for IDAP, we found significant differences between HV and CM patients at baseline as well as in the CM patients before and after the GON-B (repeated measure ANOVA F(1,34) = 12.51, *p* = 0.001). After post-hoc comparison we found that CM patients differed from HV by a steeper IDAP slope at baseline, suggesting a lower serotonin tone (CM: 0.76 ± 0.95 vs. HV: 0.03 ± 0.76; *p* = 0.01)(see Table 1). One week after the GON-B (T1) the baseline steep IDAP slope flattened and reached negative values, indicating an inversion in serotonergic activity (pre GON-B slope: 0.76 ± 0.96 vs. post GON-B: − 0.17 ± 0.93, *p* < 0.0001).

**Fig. 3:**
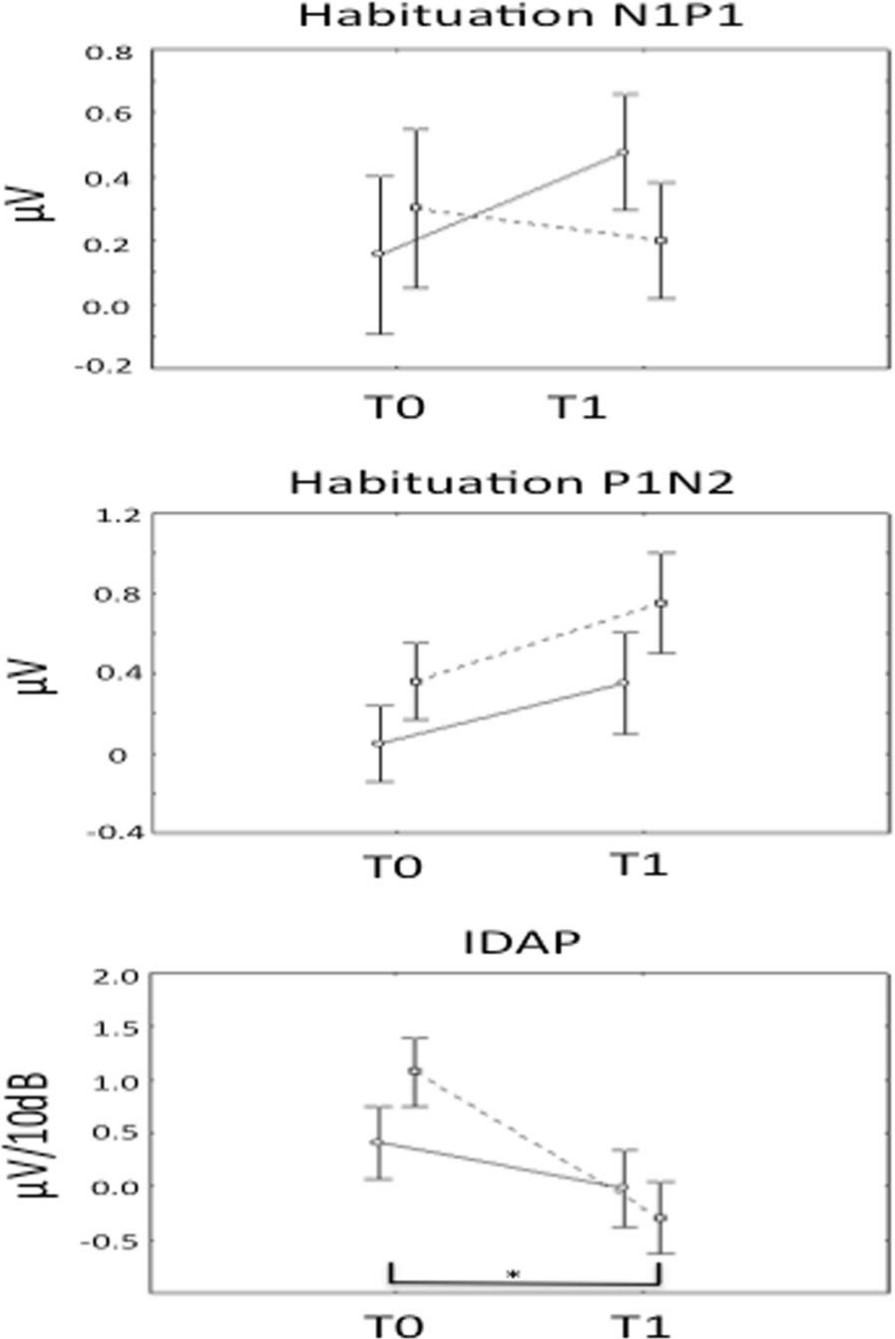
Responders vs. No-responders difference in early neurophysiological responses after GON-B. One week after the GON-B responders had no change in N1P1 response. For P1N2 component of habituation both Responders and Non-responders had a trend towards the reduction of habituation degree (although not significant, *p* = 0.11). No significant difference was found after multiple comparison tests. On the other hand, CM patients had an early reduction in IDAP slope, corresponding to an increase in serotonin firing, after GON-B (*p* = 0.008), with a significant difference at multiple comparison tests in Responders vs. Non-responders (*p* = 0.004). Responders are in dotted, No-responders in continuous line

In the sub-analysis, repeated-measures ANOVA showed a difference in neurophysiological parameters between responders and non-responders after GON-B. Although habituation of N1P1 and P1N2 components did not differ neither for time or group (see Table 2), IDAP slope showed a significant decrease only in the responder group (*p* = 0.004) (see Fig. 3).

**Table 2:** Time effect of neurophysiological parameters between Responders and Non-responders

Variables	Effect	SS	DoF	F value	*p* value
Habituation of N1P1	GROUP	0.03	1	0.08	0.79
	TIME	0.10	1	0.32	0.58
	TIME*GROUP	0.36	1	1.17	0.30
Habituation of P1N2	GROUP	1.01	1	2.12	0.17
	TIME	0.97	1	2.90	0.11
	TIME*GROUP	0.02	1	0.05	0.83
IDAP	GROUP	0.28	1	0.25	0.63
	TIME	6.02	1	9.66	0.008°
	TIME*GROUP	1.67	1	2.68	0.13

The symbol ° indicates the level of significance obtained by the repeated measures model

### Correlations between neurophysiological and clinical changes at the group level

**Fig. 4:**
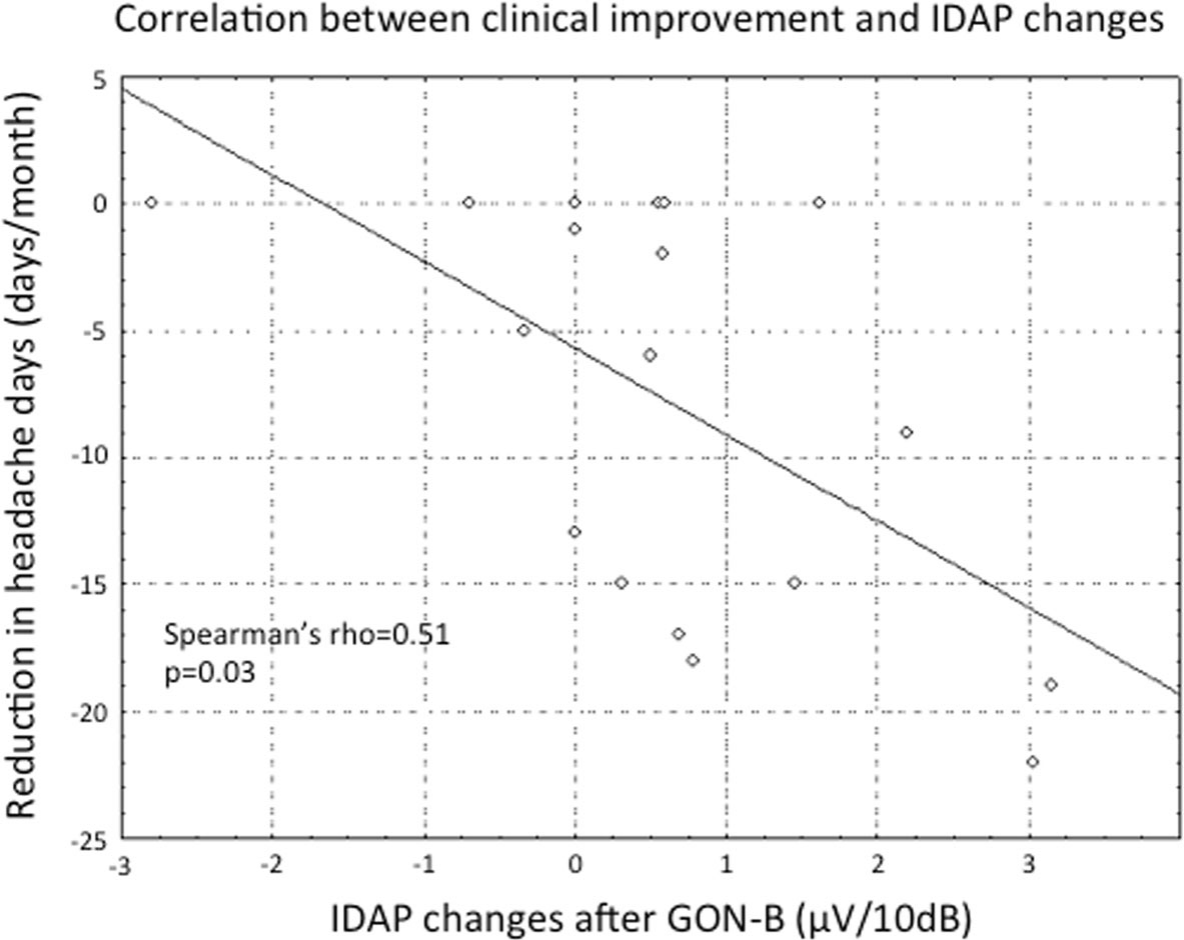
Correlation analysis between clinical improvement and IDAP changes. At the group level, the reduction in IDAP value was positively correlated with the reduction of day of headache in the follow-up month. Positive IDAP values indicate that the T1 measurement was smaller than the first. The calculation for IDAP change was: IDAP change = IDAP(T0)-IDAP(T1). On the other hand for headache days calculation was: Headache reduction = days(T2)-days(T0)

Furthermore, the reduction in headache days after treatment (from T0 to T2) correlated positively with the magnitude of the change in the slope of the IDAP between T0 and T1. The bigger was the flattening of IDAP slope (i.e. the higher serotonin firing), the better was the clinical response (Spearman’ rho = 0.51, *p* = 0.03) (see Fig. 4). There was no correlation between the baseline IDAP slope and the reduction in headache days after GON-B (Spearman’ rho = 0.31, *p* = 0.25).

### Analysis of possible sources of bias

To search for possible confounding factors in our results, we searched possible differences between CM patients with and without MOH, and between responders and non-responders. VEP Habituation did not differ between CM patients with or without MOH for N1P1 (H = 0.93; *p* = 0.34) or P1N2 (H = 0.93; *p* = 0.34) components. As well, IDAP value didn’t differ between CM with and without MOH (H = 0.27, *p* = 0.60). Analysing responders and non-responders using the Kruskal-Wallis test, we found no difference in age (H = 1.34; *p* = 0.25), number of anti-migraine acute medication intake (H = 0.47; *p* = 0.49), presence of MOH (Fisher 2 × 2 test = 0.00; df = 1; *p* = 0.13), number of headache days (H = 1.57; *p* = 0.21), VEP N1P1 habituation (H = 0.01; *p* = 0.92), P1N2 (H = 1.10; *p* = 0.29) and IDAP slopes (H = 1.93; *p* = 0.16).

## Interpretation of results

Due to the low rate of response and the need to treat patients for a long time before having any clinical feedback about the efficacy of the treatment itself, having an early predictive tool to identify if a specific treatment is effective or not for a given patient is valuable information from a medical, social and economic point of view. In this study we tested if a greater occipital nerve block (GON-B) could be such a tool by assessing its long-term clinical efficacy and its short effects on brain physiology.

We found that a single GON-B produced after 1 month a clinical benefit with a 34% reduction in headache days and a return to episodic migraine in 50% of the cases. In addition, we showed that this beneficial effect was associated with a decrease of intensity dependence of auditory evoked potentials (IDAP) 1 week after the block in clinical responders, suggestive of an increase in central serotonergic tone.

To our knowledge, this study is the first to examine the correlation of serotonergic tone, indexed by IDAP, with CM and in particular its variation in association with clinical improvement. We found that patients with CM had a low baseline serotonergic tone, which increased after a clinically successful GON-B. This occurred especially in those patients who switched from a chronic form of migraine to an episodic one. Furthermore, the degree of IDAP flattening after 1 week was directly correlated to the improvement of migraine in terms of reduction of headache days at 1 month post-GON-B. This result may thus be of some utility for clinicians who plan to perform a GON-B and like to have an early feedback of the long-term efficacy of GON-B.

The change in IDAP is not unexpected because of its strong correlation with central serotonin pathways. Migraine in general is considered since a long time to be a low serotonin disorder and in CM in particular serotonin levels are thought to be even lower on the basis of clinical (e.g. high comorbidity with depression) and biochemical data [31, 32]. Contrary to the IDAP abnormality in CM, we also found a normal pattern of VEP habituation in CM with or without MOH, which is consistent with previous findings and with the hypothesis that CM is a sort of a “never ending attack” [21, 33].

VEP habituation returns to the normal non-habituation pattern of episodic migraines, when patients with CM are successfully treated [24]. In line with these results, we found that habituation decreased for the PR-VEP N1P2 component with clinical improvement so that at T1 it differed from that of HV.

In previous studies of episodic migraineurs abnormal IDAP and VEP habituation were thought to be linked interictally because decreased habituation of auditory potentials may lead to a steep IDAP slope and low central serotonin activities could explain both abnormalities [26] .

A possible explanation for the dissociation between IDAP and VEP habituation changes in our study could be that in chronic migraine the apparently normal habituation pattern is due to changes in brain plasticity more than to a simple change in serotonergic tone [34, 35]. Chronic migraine is characterized by persistent hyperexcitability of sensory cortices [22], suggesting that the repetition of attacks induces plastic alterations of the excitatory-inhibitory balance that is fundamental for tuning synaptic plasticity and circuitry [36]. In this concept, inhibitory responses tend to decrease more than excitatory ones following stimulus repetition [37] and are also negatively modulated by neurotransmitters, such as acetylcholine, noradrenalin and oxytocin. The resulting disinhibition might thus promote hyperesponsivity [36].

In the presence of reduced inhibitory activity, a higher cortical preactivation level might be achieved and supported by a low serotonin activity. For this reason, the pattern of a normal habituation found in CM may actually be a “pseudo-normal habituation”, related to LTD-mediated inhibitory responses more that just a “ceiling effect” as hypothesized for episodic migraine [38] and lately validated by several consistent experiments (see [17] for a review). LTD-mediated inhibition responses were found more commonly in CM and in EM with high attack frequency than in episodic migraineurs with a low frequency of attacks [39].

In patients reversing from a chronic to an episodic pattern of migraine, we observed an early flattening of the IDAP response after the GON-B, indicating that clinical improvement is likely to be associated with an increase in serotonergic firing.

Although the precise mechanism of action of GON-B is unknown, it is thought to modulate brain excitability acting on input gate at the brainstem level. Although cervical stimulation has been shown to increase directly brain serotonin [40, 41], such evidence is lacking for GON-B.

The IDAP slope flattening, and thus probably central serotonin activity, positively correlated with the reduction of total headache days after a month at group level, reinforcing a possible role of serotonin in remission from CM. Both at cortical and thalamic levels, serotonin is able to modulate the excitatory/inhibitory balance, both directly and by modifying the action of other neurotransmitters like dopamine [42–44]. Serotonin-related metaplasticity, characterized by a shift from inhibitory to excitatory activity, was already found in the hippocampus [45]. Taken together, these results corroborate the idea that serotonin may be a crucial actor in the plastic brain changes that accompany migraine chronification and reversal. In our study we provide indirect evidence for such a role, which needs to be confirmed by more direct assessments of central serotonergic neurotransmission.

## Conclusions

A single greater occipital nerve block showed a good clinical response in chronic migraine patients with or without acute medication overuse. Clinical improvement, and in particular the reversal to an episodic migraine pattern was significantly associated with a decrease of intensity dependence of auditory evoked potentials (IDAP) one week after the block. As IDAP is thought to reflect central serotonergic activity and it is in addition increased at baseline in chronic migraineurs, it is likely that serotonin plays a crucial role in migraine chronification and associated plastic brain changes.

## Abbreviations

ASF: amplitude/stimulus intensity function
CM: Chronic Migraine
EM: episodic migraine
GON: Greater Occipital Nerve
GON-B: Greater Occipital Nerve Block
HV: Healthy Volunteers
IDAP: intensity dependence of auditory evoked potentials
LTD: long term depression
MOH: Medication Overuse Headache
NR: Non-responders
PR-VEPs: Pattern reversal-Visual evoked potentials
R: Responders
VEP: visual evoked potentials
